# Structural mechanism of SGLT1 inhibitors

**DOI:** 10.1038/s41467-022-33421-7

**Published:** 2022-10-28

**Authors:** Yange Niu, Wenhao Cui, Rui Liu, Sanshan Wang, Han Ke, Xiaoguang Lei, Lei Chen

**Affiliations:** 1 State Key Laboratory of Membrane Biology, College of Future Technology, Institute of Molecular Medicine, Peking University, Beijing Key Laboratory of Cardiometabolic Molecular Medicine, Beijing, China; 2 National Biomedical Imaging Center, Peking University, Beijing, China; 3 Taishan College, Shandong University, Qingdao, China; 4 Peking-Tsinghua Center for Life Sciences, Peking University, Beijing, China; 5 Beijing National Laboratory for Molecular Sciences, Key Laboratory of Bioorganic Chemistry and Molecular Engineering of Ministry of Education, Department of Chemical Biology, College of Chemistry and Molecular Engineering, Synthetic and Functional Biomolecules Center, Beijing, China

**Keywords:** Cryoelectron microscopy, Pharmacology, Diabetes, Membrane proteins, Permeation and transport

## Abstract

Sodium glucose co-transporters (SGLT) harness the electrochemical gradient of sodium to drive the uphill transport of glucose across the plasma membrane. Human SGLT1 (hSGLT1) plays a key role in sugar uptake from food and its inhibitors show promise in the treatment of several diseases. However, the inhibition mechanism for hSGLT1 remains elusive. Here, we present the cryo-EM structure of the hSGLT1-MAP17 hetero-dimeric complex in the presence of the high-affinity inhibitor LX2761. LX2761 locks the transporter in an outward-open conformation by wedging inside the substrate-binding site and the extracellular vestibule of hSGLT1. LX2761 blocks the putative water permeation pathway of hSGLT1. The structure also uncovers the conformational changes of hSGLT1 during transitions from outward-open to inward-open states.

## Abstract (web summary)

SGLT1 is essential for glucose, galactose and water uptake from the intestine, and its inhibitors have broad therapeutic potential. Here, the authors describe the cryo-EM structure of human SGLT1 in complex with an inhibitor.

## Introduction

SGLT are sodium-coupled transporters that uptake glucose into the cell together with sodium ions^1^. Two members of SGLT family proteins, SGLT1 and SGLT2, play important physiological roles in humans^1,2^. As the founding member of SGLT family transporters, SGLT1 is encoded by the *SLC5A1* gene which is mainly expressed in the gastrointestinal tract and kidney^3^. SGLT1 is essential for glucose, galactose and water uptake from the intestine and the genetic loss-of-function mutations of the human *SLC5A1* gene lead to intestinal glucose galactose malabsorption^1^. SGLT2 is encoded by the *SLC5A2* gene which is mainly expressed in the kidney and is responsible for the reabsorption of the majority of glucose from glomerular filtrate^1,4^. The inactivation mutations of the human *SLC5A2* gene cause familial renal glucosuria^1^. The differential biological functions, tissue distributions, and drug sensitivities between SGLT1 and SGLT2 are exploited for the treatment of related human diseases. Blocking the function of SGLT2 could enhance glucose excretion through urine and is widely used for the treatment of diabetes^5^. Recent clinical trials suggest that dual inhibition of both SGLT1 and SGLT2 might have advantages in type 2 diabetes patients compared with inhibition of SGLT2 alone^6^. Moreover, SGLT1-specific inhibitors have therapeutic potential for the treatment of constipation^7^. Furthermore, it is reported that SGLT inhibitors might be useful for the diagnosis and treatment of certain types of cancers^8,9^.

Current available SGLT inhibitors are small molecules developed based on the natural product phlorizin. According to their selectivity, these compounds can be divided into three groups: SGLT2-specific inhibitors, such as empagliflozin, dapagliflozin, and canagliflozin, have >100 folds selectivity for hSGLT2 over hSGLT1; SGLT1-specific inhibitors, such as KGA-2727 and mizagliflozin; dual inhibitors, such as sotagliflozin and LX2761, inhibit both SGLT1 and SGLT2 with high affinity (Supplementary Table 1)^10^.

Recent advances in the structure determination of human SGLT1 (hSGLT1) and hSGLT2 have provided initial views of their architectures^11,12^. The structure of a consensus-mutated hSGLT1 protein was captured in a nanobody-bound inward-facing apo state and molecular dynamic simulation suggests the mechanism of sugar substrate selectivity^11^. The structure of the hSGLT2-MAP17 in complex with empagliflozin in an outward-open state (hSGLT2_outward-open_) details the protein-drug interactions^12^. Despite this progress, the structure of hSGLT1 in the outward-open conformation (hSGLT1_outward-open_) is unknown, and how inhibitors inhibit SGLT1 remains elusive, which hampers the comprehensive understanding of the pharmacology of SGLT inhibitors. In this work, we present the cryo-EM structure of hSGLT1 in complex with a non-selective inhibitor LX2761, which has the highest potency among available SGLT1 inhibitors^10^.

## Results

### LX2761 locks hSGLT1-MAP17 complex in the outward-open conformation

**Fig. 1:**
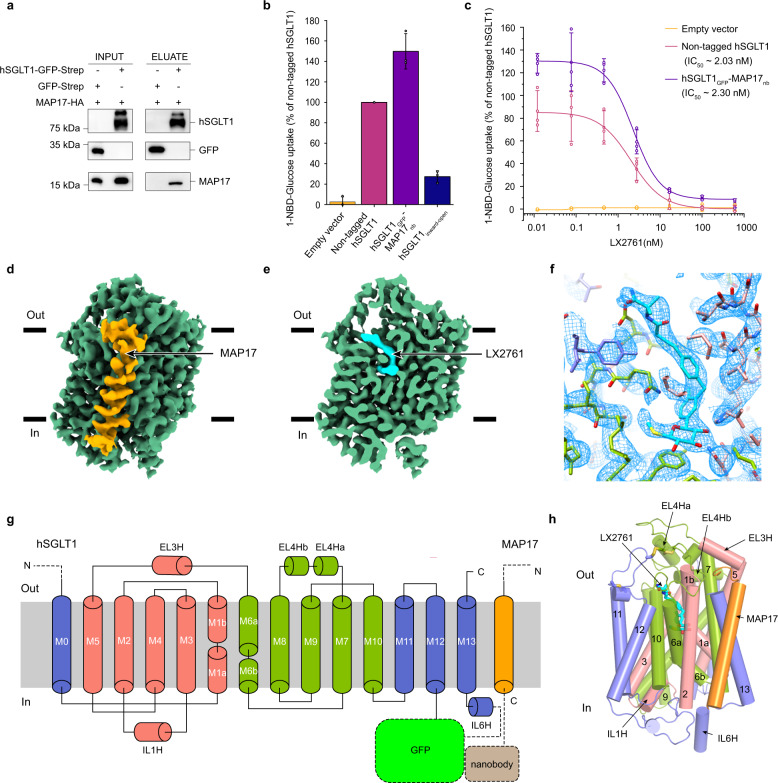
Cryo-EM structure of human SGLT1-MAP17 complex. **a** Immunoblot analysis for hSGLT1 and MAP17 in strep affinity precipitates (The experiment have been repeated with three times with similar results). **b** The 1-NBD-glucose uptake activities of non-tagged hSGLT1, hSGLT1_GFP_-MAP17_nb_, and hSGLT1_inward-open_ constructs (data are shown as means ± standard deviations; *n*  =  3 biologically independent experiments). **c** The inhibition of non-tagged hSGLT1 and hSGLT1_GFP_-MAP17_nb_ constructs by LX2761 in the 1-NBD-glucose uptake assay. The estimated IC_50_ values of LX2761 are 2.03 nM for non-tagged hSGLT1 and 2.30 nM for hSGLT1_GFP_-MAP17_nb_ (Data are shown as means ± standard deviations; *n*  =  3 biologically independent experiments). **d** Cryo-EM density map of the hSGLT1_GFP_-MAP17_nb_ complex prepared in Chimera X-1.2.5. hSGLT1 is colored in green. MAP17 is colored in orange. The detergent micelle has been omitted for clarity. **e** The cut-open view of the hSGLT1_GFP_-MAP17_nb_ complex shows the binding site of LX2761 inside hSGLT1. LX2761 is colored in cyan. The hSGLT1 is colored the same as in **d**. **f** The electron density of LX2761 and nearby residues. **g** Topology of the hSGLT1-MAP17 complex. The unsolved regions are shown as dashed lines. **h** The hSGLT1-MAP17 complex in cartoon representation, colored the same as in **g**. Helices are shown as cylinders. LX2761 is shown as sticks. Source data are provided as a Source Data file.

It is reported that over-expression of hSGLT1 in the heterologous system such as oocytes conferred robust glucose transport, suggesting hSGLT1 protein is functional by itself^13^. This is in contrast to hSGLT2, which requires MAP17 as an essential auxiliary component^14^. The structure of the hSGLT2-MAP17 complex clearly shows that the M13 of hSGLT2 interacts with MAP17^12^. In addition, MAP17-interacting residues on M13 are highly conserved between hSGLT2 and hSGLT1 (Supplementary Fig. 1a), indicating the possibility that MAP17 could also interact with hSGLT1. Indeed, our in vitro pull-down assay shows that hSGLT1 has strong interaction with MAP17 (Fig. 1a). Therefore, the hSGLT1-MAP17 complex would have the same transmembrane helices topology as the hSGLT2-MAP17 complex. Based on these findings, we applied the three-joint-tethering strategy developed for the hSGLT2-MAP17 complex to the hSGLT1-MAP17 complex^12,15^. In detail, we fused GFP between Q581 and E582 of IL6 of hSGLT1 and fused the anti-GFP nanobody after E57 of MAP17. The resulting complex (hSGLT1_GFP_-MAP17_nb_) showed robust 1-NBD-glucose uptake and inhibition by LX2671 (Fig. 1b, c), confirming that hSGLT1_GFP_-MAP17_nb_ is suitable for studying the structure and inhibition mechanism of hSGLT1. The protein of hSGLT1_GFP_-MAP17_nb_ was reconstituted into nanodisc (Supplementary Fig. 1b, c) and supplemented with LX2671 for cryo-EM studies. We obtained a map with an averaged resolution of 3.20 Å (Fig. 1d–f, Supplementary Figs. 2, 3 and Supplementary Table 2). In addition, we noticed that the protein used for structural determination of hSGLT1 in the inward-open state has a short tail after protease cleavage^11^, so we added this tail to hSGLT1 to generate the hSGLT1_inward-open_ construct and found that hSGLT1_inward-open_ has very low 1-NBD-glucose uptake (Fig. 1b), indicating this short tail might inhibit the transporting activity or the plasma membrane localization of hSGLT1.

The structure of the hSGLT1-MAP17 is overall similar to that of the hSGLT2-MAP17 (Fig. 1g, h and Supplementary Fig. 4)^12^, with a root-mean-square deviation of 0.782 Å. MAP17 binds at the peripheral and interacts with M13 of hSGLT1 (Fig. 1g, h and Supplementary Fig. 4a–c). hSGLT1 shares all of the eukaryotic SGLT-specific features observed in the hSGLT2-MAP17 structure^16^, including the extracellular shelter stabilized by three disulfide-bonds and structures packed by intracellular loops (Fig. 1h). We observed that the strong density of LX2671 extends from the sugar substrate binding sites to the extracellular vestibule of hSGLT1, reaching the extracellular solution (Fig. 1e, f). On the other hand, the intracellular gate is tightly sealed (Fig. 1e). Therefore, the structure of the hSGLT1-MAP17 in complex with LX2761 represents the outward-open conformation.

### The LX2671 binding site of SGLT1

**Fig. 2:**
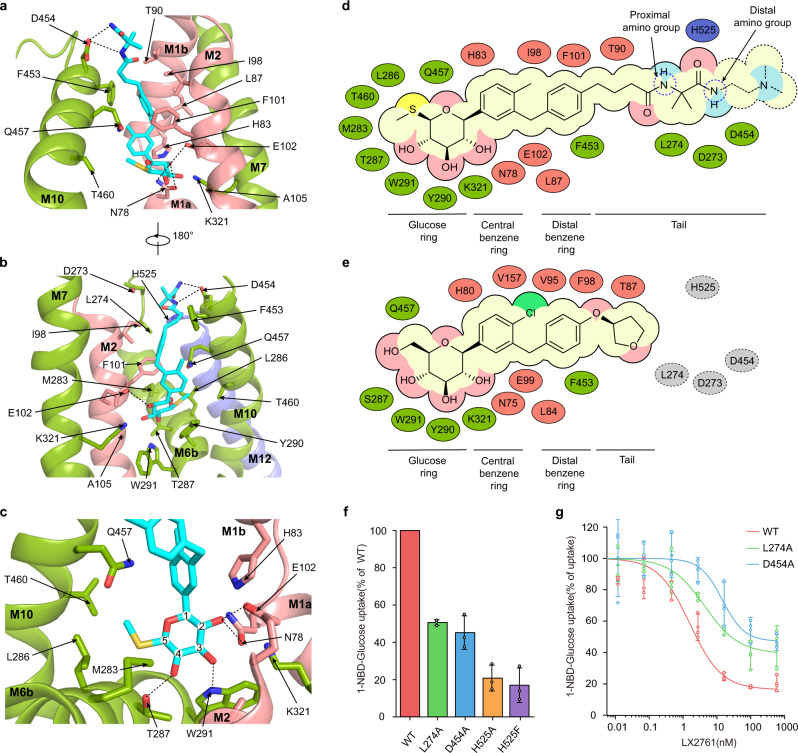
The LX2761 binding site of hSGLT1. **a** The close-up view of LX2761 binding pocket of hSGLT1. LX2761 and binding-site residues are shown as sticks. Putative hydrogen bonds are indicated as black dashed lines. **b** A 180° rotated view of **a**. **c** Interactions between hydroxyl groups of LX2761 and hSGLT1. Putative hydrogen bonds are depicted as black dashed lines. **d** Cartoon representation of the interactions between LX2761 and hSGLT1. **e** Cartoon representation of the interactions between empagliflozin and hSGLT2. The residues of hSGLT2 that might interact with LX2761 tail are colored in gray circles. **f** The 1-NBD-glucose uptake activities of LX2761 binding site mutants of hSGLT1 (Data are shown as means ± standard deviations; *n*  =  3 biologically independent experiments). **g** The inhibition of wild-type hSGLT1 and various mutants by LX2761 in the 1-NBD-glucose uptake assay (Data are shown as means ± standard deviations; *n*  =  3 biologically independent experiments). Source data are provided as a Source Data file.

LX2761 binds inside hSGLT1 via a similar pose to that observed for empagliflozin to hSGLT2^12^. LX2761 interacts extensively with hSGLT1 (Fig. 2), in agreement with its high affinity. The glucose ring of LX2761 binds at the sugar substrate site of hSGLT1 and the aglycon group extends into the extracellular vestibule (Fig. 2a–c). In detail, at the sugar ring of LX2761, the C2-OH binds to N78 on M1, E102 on M2, and K321 on M7; C3-OH binds to the side chain of W291 on M6, and main-chain carbonyl group of F101 on M2; C4-OH interacts with side chains of T287 and W291 on M6; the methylsulfanyl group at the C5 position makes hydrophobic interactions with T460 on M11, L286, and M283 on M6 (Fig. 2a–c). The central benzene ring is sandwiched between H83 on M1 and Q457 on M10 (Fig. 2a). The distal benzene ring makes hydrophobic interactions with I98 and F101 on M2 (Fig. 2a). LX2761 has a long tail that is rich in polar groups (Fig. 2d), but the density of its terminal dimethylamino group is missing, probably due to its flexibility. On the stable portion of the LX2761 tail, the dimethyl group makes hydrophobic interactions with L274 on top of M6 (Fig. 2b). The distal amino group of amide bond on the tail interacts with D454 on M10 and its adjacent carbonyl group interacts with H525 on top of M12 (Fig. 2b). Because the major difference between LX2761 and its shorter analog sotagliflozin is its longer tail, which enhances the potency towards hSGLT1 for around 20 folds (Supplementary Table 1), we want to understand the underlying basis. To evaluate the importance of these tail-interacting residues, we mutated them into other residues: L274A, D454A, H525A, and H525F. H525A or H525F mutations decreased the activity of hSGLT1 too much so that we could not reliably measure the potency of LX2761 on them (Fig. 2f). We found that both L274A and D454A decreased the potency of LX2761 (Fig. 2g), which is in agreement with our structural observation.

### The inhibitor binding pocket of hSGLT1 is larger than hSGLT2 in the center

**Fig. 3:**
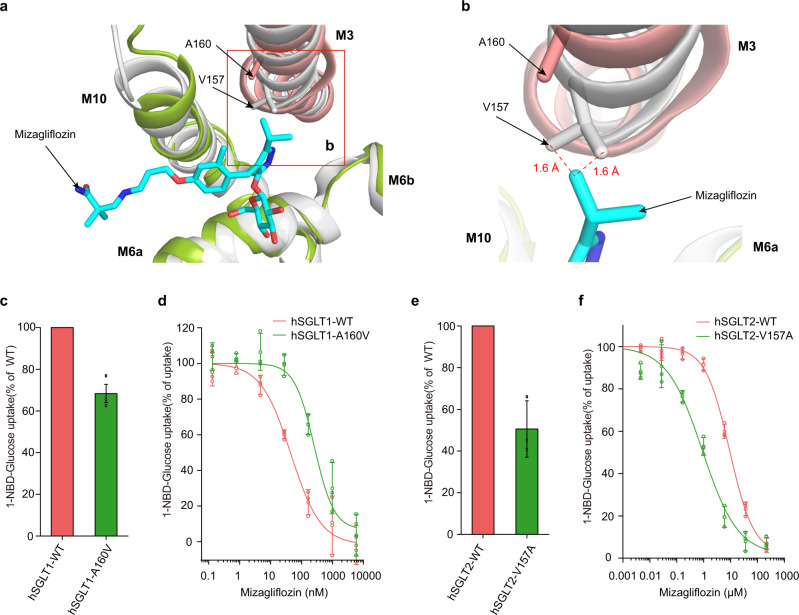
Mechanism of SGLT1-specific inhibitors. **a** The structural superposition of representative molecular dynamic simulation frame of hSGLT1_outward-open_ in complex with mizagliflozin (colored) onto the structure of hSGLT2_outward-open_ (gray, PDB ID: 7VSI). Mizagliflozin is shown as sticks. The representative simulation frame used for structural alignment is indicated in green circles shown in Supplementary Fig. 5a, b. **b** The close-up view of the sterical clashes between Mizagliflozin and V157 of SGLT2. Inter-atom distances that are smaller than their van der Waals radii are indicated by dashes. **c** The 1-NBD-glucose uptake activities of wild-type hSGLT1 and A160V mutant. **d** The inhibition of wild-type hSGLT1 and its mutant by mizagliflozin in the 1-NBD-glucose uptake assay. **e** The 1-NBD-glucose uptake activities of wild-type hSGLT2 and V157A mutant. **f** The inhibition of wild-type hSGLT2 and its mutant by mizagliflozin in the 1-NBD-glucose uptake assay. (Data are shown as means ± standard deviations; *n*  =  3 biologically independent experiments). Source data are provided as a Source Data file.

In order to elucidate the specificity of hSGLT1-specific inhibitor mizagliflozin, we compared the structure of SGLT1 with the structure of hSGLT2. We found that the shape of the binding site for the central benzene group is different between hSGLT1 and hSGLT2. The binding pocket is larger in hSGLT1 than hSGLT2 because the A160 on M3 of hSGLT1 is replaced by a bulkier V157 in hSGLT2. This would certainly result in the shrinkage of the inhibitor binding pocket in hSGLT2. To evaluate the effects of such structural difference on mizagliflozin binding, we performed molecular dynamic simulations of hSGLT1 with mizagliflozin docked into the binding site of LX2761 (Supplementary Fig. 5). Notably, the central benzene group of LX2761 is replaced by an isopropyl pyrazole group in mizagliflozin. By aligning the representative simulation frame to the structure of hSGLT2, we found the isopropyl group of mizagliflozin has sterical clashes with V157 of hSGLT2 (Fig. 3a, b and Supplementary Fig. 5c), which likely contributes to its low potency towards hSGLT2. In agreement with these observations, we found that V157A mutation of hSGLT2 enhances the potency of mizagliflozin and A160V mutation of hSGLT1 decreases the potency of mizagliflozin (Fig. 3c–f).

### Two sodium-binding sites of hSGLT1

**Fig. 4:**
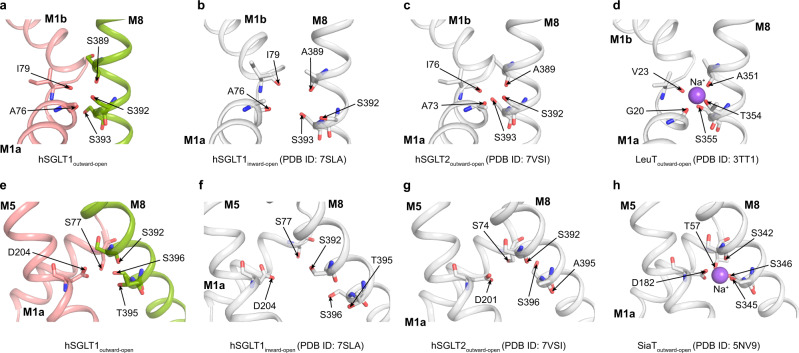
Putative sodium-binding sites of hSGLT1. **a** Putative sodium-binding site (Na2 site) of hSGLT1_outward-open_. **b** Putative sodium-binding site (Na2 site) of hSGLT1_inward-open_ (PDB ID: 7SLA). **c** Putative sodium-binding site (Na2 site) of hSGLT2_outward-open_ (PDB ID: 7VSI). **d** Putative sodium-binding site (Na2 site) of LeuT_outward-open_ (PDB ID: 3TT1). **e** Putative sodium-binding site (Na3 site) of hSGLT1_outward-open_. **f** Putative sodium-binding site (Na3 site) of hSGLT1_inward-open_. **g** Putative sodium-binding relative site (Na3 site) of hSGLT2_outward-open_. **h** Putative sodium-binding site (Na3 site) of SiaT_outward-oepn_ (PDB ID: 5NV9).

The Na^+^/glucose stoichiometry of hSGLT1 is 2:1 and there are two sodium ion binding sites in hSGLT1, namely the Na2 site and the Na3 site^16^. The Na2 site of hSGLT1_outward-open_ is supposed to be formed by side-chain hydroxyl groups of S392 and S393 on M8 and main-chain carbonyl groups of A389 on M8, A73, and I79 on M1 (Fig. 4a–d and Supplementary Fig. 4d)^16^. This site is conserved between SGLT1 and SGLT2 and is similar to the Na2 site of LeuT (PDB ID: 3TT1) (Fig. 4a–d and Supplementary Fig. 1a). Similar to the hSGLT2 structure^12^, we did not observe strong sodium density in the Na2 site of the cryo-EM map (Supplementary Fig. 4f). This site is disrupted in hSGLT1_inward-open_^11^. The Na3 site was previously observed in the outward-open structure of SiaT (PDB ID: 5NV9), which is a sialic acid transporter with 2:1 Na^+^/substrate stoichiometry^17^. The Na3 site of hSGLT1_outward-open_ is supposed to be coordinated by side chains of D204 on M5, T395 and T396 on M8, and the main-chain carbonyl group of S392 on M8 (Fig. 4e–h and Supplementary Fig. 4e)^16^. T395 in hSGLT1 is replaced by A395 in hSGLT2 (Supplementary Fig. 1a), this probably results in the loss of the Na3 site and the change of Na^+^/glucose stoichiometry in hSGLT2. In agreement with this, mutation of T395A in SGLT1 would decrease the transporting stoichiometry of sodium to glucose from 2:1 to 1:1^16^. The Na3 site is also disrupted in hSGLT1_inward-open_^11^. Although residues of both the Na2 site and the Na3 site of hSGLT1_outward-open_ are in positions, we did not observe sodium ion densities in them (Supplementary Fig. 4g), likely due to their low affinity for sodium at 0 mV^18^ or inhibitors affect their sodium affinities.

### Closed water permeation pathway

**Fig. 5:**
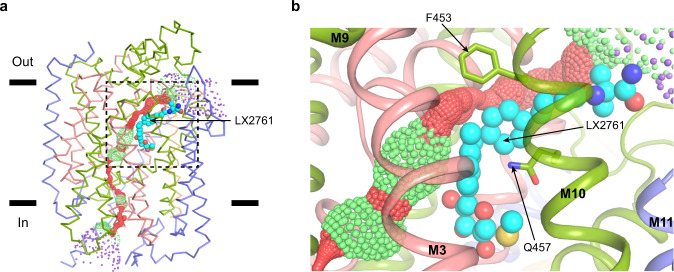
Closed water permeation pathway. **a** The estimated water permeation pathway of hSGLT1_outward-open_ is shown as small dots. The pore radii were calculated with the HOLE program. Pore radii with <2.8 Å, 2.8 Å−7.8 Å, >7.8 Å were colored as red, green and purple, respectively. LX2761 is shown as cyan spheres. **b** LX2761 blocks water permeation at the extracellular vestibule (boxed in **a**). Residues along the water permeation pathway are shown as sticks.

SGLT1 permeates to water^19^ and F453 and Q457 on M10 are along the water permeation pathway of SGLT1^20^. Recent molecular dynamic simulation based on the structure of hSGLT1_inward-open_ revealed a putative water pathway that connects the extracellular side to the intracellular side of hSGLT1^11^. We calculated the radius of the putative water pathway in the current SGLT1_outward-open_ structure, showing the pathway is blocked by the intracellular gate on one side and also blocked by LX2761 on the other side (Fig. 5). These observations are consistent with the results showing inhibitors could abolish water permeation through SGLT1^19,20^.

### Conformational changes from outward-open to inward-open state

**Fig. 6:**
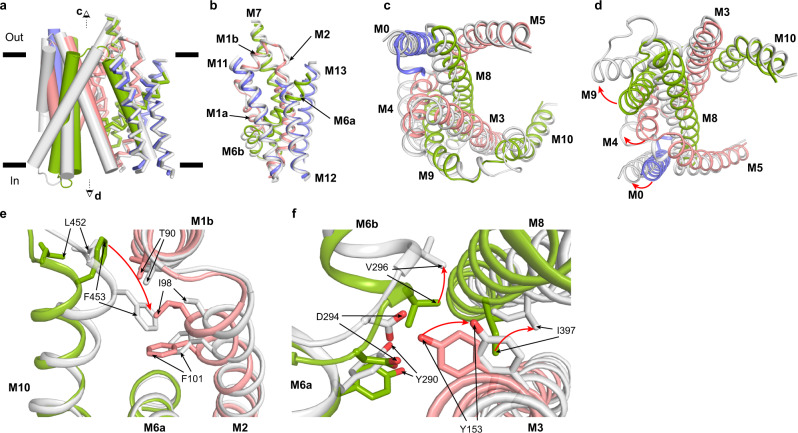
Conformational changes from outward-open to inward-open. **a** Superposition of hSGLT1_outward-oepn_ (colored) and hSGLT1_inward-open_ (gray, PDB ID: 7SLA). Helices are shown as cylinders. **b** The structural alignment of the constant module. **c** Top view of the moving module. **d** Bottom view of the moving module. The movement of helices are shown as red arrows. **e** The conformational changes of the hSGLT1 extracellular gate. **f** The conformational changes of the hSGLT1 intracellular gate. Residues which moves are shown as sticks, the movement of residues are shown as red arrows.

The availability of hSGLT1 in both inward-open and outward-open conformations allowed us to visualize the conformational changes from the outward-open state to the inward-open state (Fig. 6a). The overall structural alignment shows that half of hSGLT1, including M1, M2, M6, M7, M11, M12, and M13 are relatively stable (Fig. 6a, b), and we name these helices as the constant module. In contrast, the remaining half, including M0, M3, M4, M5, M8, M9, M10 of hSGLT1, shows larger structural changes (Fig. 6a, c, d), we name these helices as the moving module. The orchestrated movements of both modules close the extracellular gate but open the intracellular gate of hSGLT1. Particularly, in the extracellular vestibule, the upper region of M10 moves inward (Fig. 6e). F453 on M10 forms hydrophobic interactions with F101 on M2, I98 on M2, and T90 on M1 (Fig. 6e). These residues close the extracellular gate. In the intracellular vestibule, the M3, M6, and M8 move outward (Fig. 6f). Moreover, the IL6H is disordered in the inward-open state. These structural changes lead to the opening of the intracellular gate, which was sealed by residues, including I397 on M8, Y153 on M3, V296, D294 and Y290 on M6 (Fig. 6f).

## Discussion

The rich pharmacological data on various SGLT inhibitors (Supplementary Table 1) and the cryo-EM structures of hSGLT1 in complex with LX2761 and hSGLT2 in complex with empagliflozin allow us to dissect the mechanism for inhibitor binding. We found the modifications on the sugar ring, the extension of the inhibitor tail, and the replacement of the central benzene group all affect the potency and selectivity of inhibitors (Supplementary Table 1). Comparison between sotagliflozin and dapagliflozin provides insights into the effect of sugar ring modification. The hydroxylmethyl group at the C5 position of the sugar ring of dapagliflozin is substituted by a methylsulfanyl group in sotagliflozin (Supplementary Table 1). Based on our structures, this modification results in the loss of putative hydrogen bonding between C6-OH of dapagliflozin and Q457 of hSGLT1 on one hand, but the formation of hydrophobic interactions between the methylsulfanyl group of sotagliflozin and T460, L286, and M283 of hSGLT1 on the other hand, leading to the net increase of potency from 1,400 nM (dapagliflozin) to 36 nM (sotagliflozin) towards hSGLT1 (Supplementary Table 1). However, this modification has little effect on the potencies of dapagliflozin and sotagliflozin towards hSGLT2 (Supplementary Table 1). Residues of hSGLT2 that are supposed to be close to the methylsulfanyl group on the glucose ring of sotagliflozin are S460, V286, and L283, all of which are smaller than their counterparts in hSGLT1 and would presumably be less favorable for hydrophobic interactions with sotagliflozin, in agreement with the similar potencies of sotagliflozin and dapagliflozin towards hSGLT2. Comparison between LX2761 and sotagliflozin shows that the extension of the inhibitor tail enhances the affinity of LX2761 towards hSGLT1 and our mutagenesis data on residues that interact with the inhibitor tail (L274A and D454A) are in agreement with this (Fig. 2g). Notably, L274 and D454 are also conserved in SGLT2 and would also interact with the tail of LX2761 (Fig. 2e). However, the potencies of LX2761 and sotagliflozin towards SGLT2 are similar (Supplementary Table 1) and the exact reason remains elusive. The SGLT1-specific inhibitor mizagliflozin has an isopropyl pyrazole group in the center. Our structural analysis of mizagliflozin binding site suggests that the protruded isopropyl group generates sterical clashes with V157 of hSGLT2, contributing to the selectivity of mizagliflozin. Our structures suggest that all of the currently available SGLT inhibitors likely lock the transporters in the outward-open conformation. These structural and functional data lay the foundation for further rational development of SGLT inhibitors.

Finally, structural comparison and analysis reveal the conformational changes of hSGLT1 from inward-open to outward-open state. To our surprise, we did not find large conformational changes of M1, M2, M6, and M7 as previously observed in LeuT^21,22^. Instead, we found the constant module of hSGLT1 is relatively stable but the moving module has large conformational changes during transporting cycle. The similar conformational transition was previously proposed based on the modeling studies and double electron-electron resonance measurements for vSGLT^23^. This observation highlights the diverse structural mechanisms of sodium-coupled transporters that share the common LeuT-like fold.

## Methods

### Cell culture

Sf9 insect cells (Thermo Fisher Scientific) were cultured in Sf-900 III serum-free medium (Thermo Fisher Scientific) or SIM SF serum-free medium (Sino Biological) at 27 °C. HEK293F suspension cells (Thermo Fisher Scientific) were cultured in FreeStyle 293 medium (Thermo Fisher Scientific) or SMM 293-TI medium (Sino Biological) supplemented with 1% fetal bovine serum (FBS) at 37 °C with 6% CO_2_ and 70% humidity. AD293 adherent cells (Agilent Technologies) were cultured in Dulbecco’s Modified Eagle Medium (Gibco) supplemented with 10% FBS at 37 °C with 5% CO_2_. The cell lines were routinely checked to be negative for mycoplasma contamination but have not been authenticated.

### Protein expression, purification and nanodisc reconstitution

The cDNA of human SGLT1 was cloned into the modified BacMam expression vector for screening by fluorescence-detection size-exclusion chromatography (FSEC) on a Superose 6 increase 5/150 GL column (GE Healthcare). According to the hSGLT2-MAP17 complex structure determination^12^ and conservation between SGLT1 and SGLT2, we fused a GFP containing two strep tags into IL6 of hSGLT1, between Q581 and E582 (Supplementary Table 4). We also fused the nanobody against GFP^24^ (PDB ID: 3K1K) to MAP17 C-terminal truncated construct (1–57) (Supplementary Table 4). The expression cassettes of hSGLT1 and MAP17 were further merged into one bicistronic plasmid by the LINK sequence on the vector^25,26^ for protein expression using the BacMam system^27^. For protein expression, HEK293F cells cultured in SMM 293-TI medium at a density of 2.5$\times {10}^{6}$ cells per ml were infected with 10% volume of P2 virus. Sodium butyrate (10 mM) was added to the culture 12 h after transfection to promote protein expression, and the cells were transferred to a 30 °C incubator for another 36 h before harvesting. Cells were collected by centrifugation at 3,999 g (JLA-8.1000, Beckman Coulter) for 10 min at 4 °C, and washed with TBS buffer (20 mM Tris pH 8.0 at 4 °C, 150 mM NaCl) containing 2 μg/ml aprotinin, 2 μg/ml pepstatin, 2 μg/ml leupeptin. The cells were then flash-frozen and stored at −80 °C.

For purification, the membrane pellets corresponding to 1 liter culture were resuspended and homogenized in 15 ml TBS buffer containing protease inhibitor (2 μg/ml aprotinin, 2 μg/ml pepstatin, 2 μg/ml leupeptin, and 1 mM phenylmethanesulfonyl fluoride (PMSF)), 10 mM MgCl_2_, 0.7 μg/ml benzonase, and 10 μM LX2761. The crude membrane solution was incubated at 37 °C for 1 h for LX2761 binding. Digitonin (BioSynth) was added to a final concentration of 1% (W/V) after cooling to 4 °C, and stirred for 1 h to solubilize proteins. The insoluble debris was removed by centrifugation at 193,400 g (Ti50.2, Beckman Coulter) for 30 min. Subsequently, the supernatant was loaded onto 5 ml Streptactin Beads 4FF (Smart Lifesciences) column and washed with 50 ml wash buffer 1 (TBS buffer plus 40 μM glyco-diosgenin (GDN, Anatrace), 1 μM LX2761, 10 mM MgCl_2_ and 1 mM adenosine triphosphate (ATP)) to remove contamination of heat shock proteins. Then, the column was extensively washed with 50 ml wash buffer 2 (TBS buffer supplemented with 40 μM GDN and 1 μM LX2761). The target protein was eluted with elution buffer contained 50 mM Tris pH 8.0 at 25 °C, 150 mM NaCl, 40 μM GDN, 1 μM LX2761 and 10 mM D-desthiobiotin (IBA). The eluate was loaded onto HiTrap Q HP (GE Healthcare) and the hSGLT1-MAP17 complex was separated from aggregates with a linear gradient from 0 mM NaCl to 1000 mM NaCl in buffer containing 20 mM Tris pH 8.0 at 4 °C, 40 μM GDN, and 1 μM LX2761. The fractions containing the hSGLT1-MAP17 complex were collected for nanodisc reconstitution. MSP2X that contains two copies of MSP1E3D1 was purified according to previously described method^28^. The hSGLT1-MAP17 complex was mixed with MSP2X and soybean polar lipids extract (SPLE, solubilized in 1% (W/V) GDN, Avanti) at a molar ratio of protein: MSP2X: SPLE = 1: 4: 400. The mixture was allowed to equilibrate for 1 h at 4 °C, and then Bio-beads SM2 (Bio-Rad) was added to initiate the reconstitution with constant rotation at 4 °C. Bio-beads SM2 were added to the mixture four times within 24 h to gradually remove detergents from the system. Afterwards, the proteins that were not reconstituted in lipid nanodisc was removed by centrifugation at 86,600 g for 30 min in TLA 100.3 rotor (Beckman Coulter). The supernatant containing hSGLT1-MAP17 complex reconstituted in lipid nanodisc was loaded onto the 1 ml Streptactin Beads 4FF column to remove empty nanodisc. The elution from Streptactin Beads 4FF column was concentrated and subjected onto a Superose 6 increase 10/300 GL column (GE Healthcare) in buffer that contained 20 mM HEPES pH 7.5, 150 mM NaCl and 1 μM LX2761. The peak fractions corresponding to the hSGLT2-MAP17 complex in lipid nanodisc were collected for cryo-EM sample preparation.

### Affinity precipitation and western blotting

Plasmid contains MAP17 with C-terminal HA tag was co-transfected with GFP-strep tagged hSGLT1 or vector expressing strep tagged GFP alone. Two days post-transfection, the cells were collected and lysed in 150 μl lysis buffer containing 50 mM Tris pH 8.0 at 4 °C, 150 mM NaCl, 1% (W/V) GDN, 2 μg/ml aprotinin, 2 μg/ml pepstation, 2 μg/ml leupeptin, and 1 mM PMSF. The debris were removed by centrifugation at 86,600 g for 30 min in TLA 100.3 rotor (Beckman Coulter). The supernatant was incubated with Streptactin Beads 4FF (Smart Lifesciences) for 4 h at 4 °C. The beads were washed four times with TBS buffer containing 40 μM GDN, and eluted with 50 mM Tris pH 8.0 at 4 °C, 150 mM NaCl, 40 μM GDN and 10 mM D-desthiobiotin (IBA). The proteins were resolved by SDS–PAGE and then transferred onto polyvinylidene difluoride (PVDF) membrane (Millipore, #IPVH00010). The membrane was blocked with 5% non-fat milk in TBST buffer (20 mM Tris pH 8.0 at 4 °C, 150 mM NaCl and 0.1% Tween-20) for 1 h at 25 °C and incubated with primary antibodies (strep mouse monoclonal antibody (Earthox, #E022140-03) at a 1:5,000 dilution, HA rabbit polyclonal antibody (Cell Signaling Technology, #3724) at a 1:2,000 dilution) overnight at 4 °C. The membrane was washed four times with TBST buffer for 5 min each on a rocker at 25 °C, followed by incubation with horseradish-peroxidase (HRP) linked anti-mouse IgG (Invitrogen, #31444) or HRP linked anti-rabbit IgG (Invitrogen, #31460) at a 1:10,000 dilution in fresh blocking buffer for 1 h at 25 °C. After incubation with secondary antibodies, membrane was washed four times with TBST buffer for 5 min each on a rocker at 25 °C. Signal was detected with enhanced chemiluminescence reagents (Tanon).

### 1-NBD-glucose uptake assay

1-NBD-glucose uptake assay was a rapid and reliable method for the measurement of the potencies of SGLT inhibitors^12^. AD293 cells cultured in 12-well plates were transfected with wild-type hSGLT1 or its mutants (Supplementary Table 4). One day post-transfection, cells were seeded into 96-well plate coated with poly-D-lysine. After attachment, cells were washed with 200 μl per well PBS (10 mM Na_2_HPO_4_, 2 mM KH_2_PO_4_, 137 mM NaCl, and 2.7 mM KCl) twice, followed by incubation at 37 °C with 5% CO_2_ for 1 h in uptake buffer (10 mM HEPES pH 7.4, 150 mM NaCl, 1 mM CaCl_2_, and 1 mM MgCl_2_) supplemented with 600 μM 1-NBD-glucose and 0.3% bovine serum albumin (BSA) plus different concentration of LX2761. Subsequently, cells were washed three times with 200 μl per well PBS to stop the uptake. Then the cells were lysed with 150 μl per well TBS buffer containing 1% Triton X-100 for 30 min at 25 °C. The lysates were transferred to a clear-bottom black 96-well plate for 1-NBG-glucose detection with excitation at 445 nm, emission at 525 nm on an Infinite 200Pro imager (Tecan Life Sciences). Protein concentration in 96 well plate was determined using a BCA protein assay kit (CWBIO). The 1-NBD-glucose fluorescence signals in each well were normalized to the protein concentration. The specific 1-NBD-glucose uptake of each well was calculated by subtracting NBD fluorescence signals in wells transfected with empty vector. The specific 1-NBD-glucose uptake was plotted against the log of the concentration of inhibitors. The IC_50_ was calculated with OriginPro 2021b using the equation: *Y* = 100/($1+10^{((X-{{{\rm{LogIC}}}}_{50}))}$).

### Cryo-EM sample preparation and data collection

Surface of Quantifoil Au 300 mesh R 0.6/1.0 grids were coated with graphene oxide^29^. Aliquots of 2.5 µl of hSGLT1_GFP_-MAP17_nb_ protein in nanodisc at a concentration of ~0.1 mg/ml in the presence of 400 μM LX2761 and 0.5 mM fluorinated octyl-maltoside (FOM, Anatrace) were applied to the grids. After 60 s incubation on the grids at 4 °C under 100% humidity, the grids were then blotted for 4 s using a blot force of 4, and then plunge-frozen into liquid ethane using a Vitrobot Mark IV (Thermo Fisher Scientific). The grids were transferred to a Titan Krios electron microscope (Thermo Fisher) operating at 300 kV and a K2 Summit direct detector (Gatan) mounted post a quantum energy filter (slit width 20 eV). SerialEM-3.6.11 was used for automated data collection. Movies were recorded in super-resolution mode and a defocus range of −1.4 µm to −1.8 μm with a nominal magnification of 165,000×, resulting in a calibrated pixel size of 0.4105 Å. Each stack of 32 frames was exposed for 8 s, with an exposing time of 0.25 s per frame at a dose rate of 4.7 electrons per Å^−2^ per second.

### Cryo-EM image processing

A total of 6586 micrographs were corrected for beam-induced drift and 2x binned to a pixel size of 0.821 Å using MotionCor2^30^. The contrast transfer function parameters of dose-weighted micrographs were estimated by Gctf^31^. Micrographs were manually screened and a total of 6,366 micrographs were used for image processing. A total of 3,081,611 particles were auto-picked using Gautomatch-0.56 (developed by K. Zhang). All particles were extracted with a box size of 120 and 2x binned (pixel size 1.642 Å) in Relion-3.1^32^. These particles were subjected to two-dimensionally classification with cryoSPARC-3.1.0^33^. Two rounds of 2D classification were carried out and 957,209 particles from classes exhibiting recognizable hSGLT1 features were selected for further processing. A batch size of 50,000 particles was used to generate an initial 3D reference model. The initial model was low-pass filtered to resolution of 8 Å, 15 Å, 25 Å and 35 Å used as references for a heterogeneous refinement without symmetry imposed. Subsequently, 484,041 particles from the best classes were re-extracted using a box size of 240 at 0.821 Å per pixel and re-centered in Relion-3.1 and then refined with subsequent NU-refinement and local refinement in cryoSPARC-3.1.0, resulting in a reconstruction with overall resolution of 3.08 Å. To further improve the density of LX2761, seed-facilitated 3D classification was performed^34^. A set of 843,705 particles was enriched from seed-facilitated 3D classification in cryoSPARC-3.1.0. NU-refinement and local refinement of these particles yielded a reconstruction at 3.08 Å. Refined particles were subjected to 3D classification in Relion-3.1without alignment using a soft mask encompassing the protein and excluding the detergent micelle with *k*  =  5, and *T*  =  40. The references used in 3D classification were the 3.08 Å reconstruction low-pass filtered to 4 Å, 8 Å, 12 Å, 16 Å and 20 Å. The classes of the last 12 iterations with the best features of hSGLT1 and LX2761 in 3D classification were combined and the duplicated particles were removed. The final 12,133 particles were re-extracted using a box size of 280 and a pixel size of 0.821 in Relion-3.1. These particles were subjected to NU-refinement and local refinement in cryoSPARC-3.1.0, yielded a reconstruction at 3.20 Å. Resolution was estimated using the Fourier shell correlation (FSC) = 0.143 criterion. Local resolution was calculated using Relion-3.1.

### Model building

The homology model of the hSGLT1-MAP17 complex was generated from the hSGLT2-MAP17 complex using SWISS-MODEL server^35^. The model was fitted into the cryo-EM map using UCSF Chimera^36^ and manually adjusted using Coot^37^. LX2761 was manually fitted into the electron density. The density of 2-(dimethylamino)ethyl group of LX2761 was missing probably due to its flexibility and thus removed from the final model. The model was further refined against the map using Phenix^38^. The statistics of the model refinement are summarized in Supplementary Table 2. The water permeation pathway was calculated using HOLE^39^. Figures were generated using UCSF Chimera, Chimera X^36,40^ and PyMol.

### All-atom molecular dynamics simulations

The protein scaffold of the hSGLT1-LX2761 was used for docking calculation of SGLT1-mizagliflozin complex using Autodock Vina^41^. A grid box of 26 Å$\times$26 Å$\times$28 Å was centered on the binding site of LX2761. We used the binding pose with the lowest energy as the initial structure of the molecular dynamics. We used CHARMM-GUI to generate the system. We used the DMPC to build the phospholipid bilayer and set the concentration of NaCl to 0.15 M. We used the ff14SB force field parameter set for the hSGLT1-MAP17 complex, the gaff force field parameter set for mizagliflozin, the TIP3P model for water and the lipid14 force field parameter set for the lipid with the Leap module of the AmberTools21. Using the particle mesh Ewald method with periodic boundary conditions, long-range electrostatic effects were modeled. An 8 Å cut-off was applied to Lennard-Jones and electrostatic interactions. Three repeated Molecular dynamics simulations were performed according to the following steps: (1) Minimization for waters and lipids was performed with a maximum cycle of 100,000 and with the steepest descent algorithm for the first 50,000 cycles with the SHAKE algorithm (an algorithm for constrained molecular dynamics) inactivated. Positional restraints of 0.5 kcal/mol·Å^2^ were applied to the protein. (2) Minimization for the whole system was performed with a maximum cycle of 100,000 and the steepest descent algorithm for the first 50,000 cycles with the SHAKE algorithm inactivated. (3) Heating process was performed with a periodic boundary for constant volume with the SHAKE algorithm activated. Thus, the angle between the hydrogen atoms was fixed. The temperature increased from 0 K to 298 K with Langevin dynamics with the collision frequency of 1 ps^−1^. (4) A density equilibrium process was performed with a periodic boundary for constant pressure with anisotropic pressure scaling and a constant temperature of 298 K. (5) A 10 ns pre-equilibrium process was performed with a periodic boundary for constant pressure and constant temperature of 298 K, followed by a 500 ns equilibrium process. The representative frame was obtained by the cluster analysis tool in Chimera^42^.

### Quantification and statistical analysis

Global resolution estimations of cryo-EM density maps are based on the 0.143 Fourier Shell Correlation criterion^43^. The local resolution was estimated using Relion-3.1^32^. The number of independent reactions (N) and the relevant statistical parameters for each experiment (such as mean or standard deviation) are described in the figure legends. No statistical methods were used to pre-determine sample sizes.

### Reporting summary

Further information on research design is available in the Nature Research Reporting Summary linked to this article.

## Supplementary information

Supplementary Information

Reporting Summary

## Data Availability

The data that support this study are available from the corresponding authors upon reasonable request. The cryo-EM map has been deposited in the Electron Microscopy Data Bank (EMDB) under accession code EMD-32617 (hSGLT1-MAP17 complex). The coordinates have been in the RCSB Protein Data Bank (PDB) under accession code 7WMV [10.2210/pdb7WMV/pdb] (hSGLT1-MAP17 complex). PDB entries (3K1K [10.2210/pdb3K1K/pdb],7VSI [10.2210/pdb7VSI/pdb], 7SLA [10.2210/pdb7SLA/pdb], 3TT1 [10.2210/pdb3TT1/pdb] and 5NV9 [10.2210/pdb5NV9/pdb]) used in this study were downloaded from Protein Data Bank. The source data underlying Figs. 1a–c, 2f–g, 3b–e, Supplementary Figs. 1b–c, 2d and 5a–c are provided as a Source Data file. Source data are provided with this paper.

## Source data

Source Data
