# Supplementary material for: Structural mechanism of SGLT1 inhibitors
Source: Nat Commun. 2022 Oct 28;13:6440. doi: 10.1038/s41467-022-33421-7 (PMC9616851; doi:10.1038/s41467-022-33421-7)
Supplement: Supplementary file 1 — Supplementary Information [file 41467_2022_33421_MOESM1_ESM.pdf]

Supplementary Information for

Structural mechanism of SGLT1 inhibitors

Yange Niu, Wenhao Cui, Rui Liu, Sanshan Wang, Han Ke, Xiaoguang Lei, and Lei Chen*

* To whom correspondence should be addressed: Lei Chen (chenlei2016@pku.edu.cn)

This PDF file contains:

Supplementary Figs. 1-5

Supplementary Table 1-4

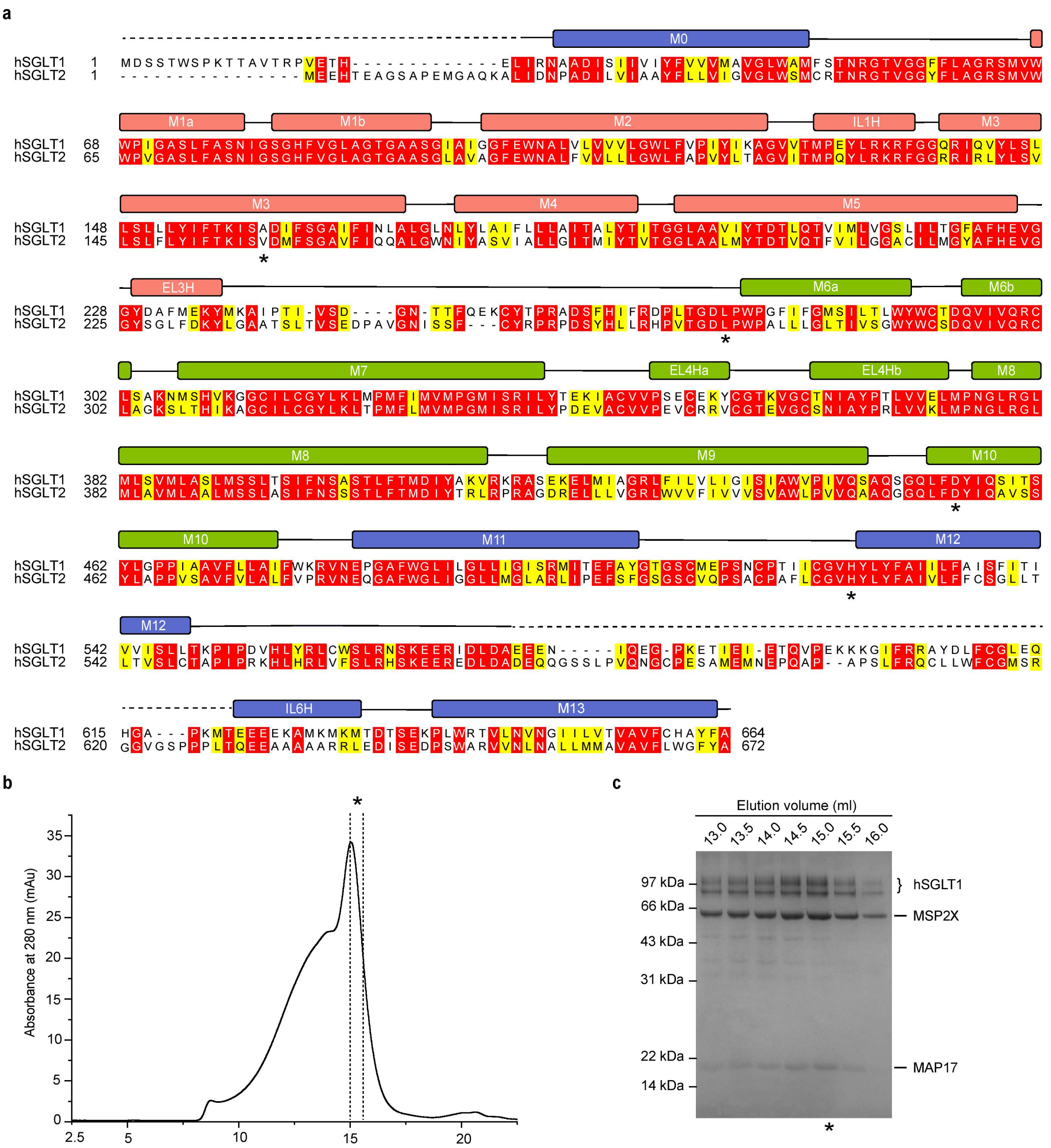

Supplementary Fig. 1 | Biochemical characterization of hSGLT1$_{GFP}$-MAP17$_{nb}$ in nanodisc. a, The sequences of hSGLT1 and hSGLT2 were aligned using MEGA-X. Similar residues are shaded in yellow and identical residues are shaded in red. The secondary structural elements are indicated above the sequence alignment. The unsolved regions are shown as dashed lines. Asterisks below denote residues selected for mutagenesis experiments. **b,** The gel filtration profile of hSGLT1 in nanodisc. Fractions between dashed lines indicated by asterisk were used for cryo-EM sample preparation. **c,** SDS-PAGE analysis of the purified hSGLT1 in nanodisc. Asterisk indicates the fraction for cryo-EM sample preparation. Experiment was repeated independently more than three times with similar results. Source data are provided as a Source Data file.

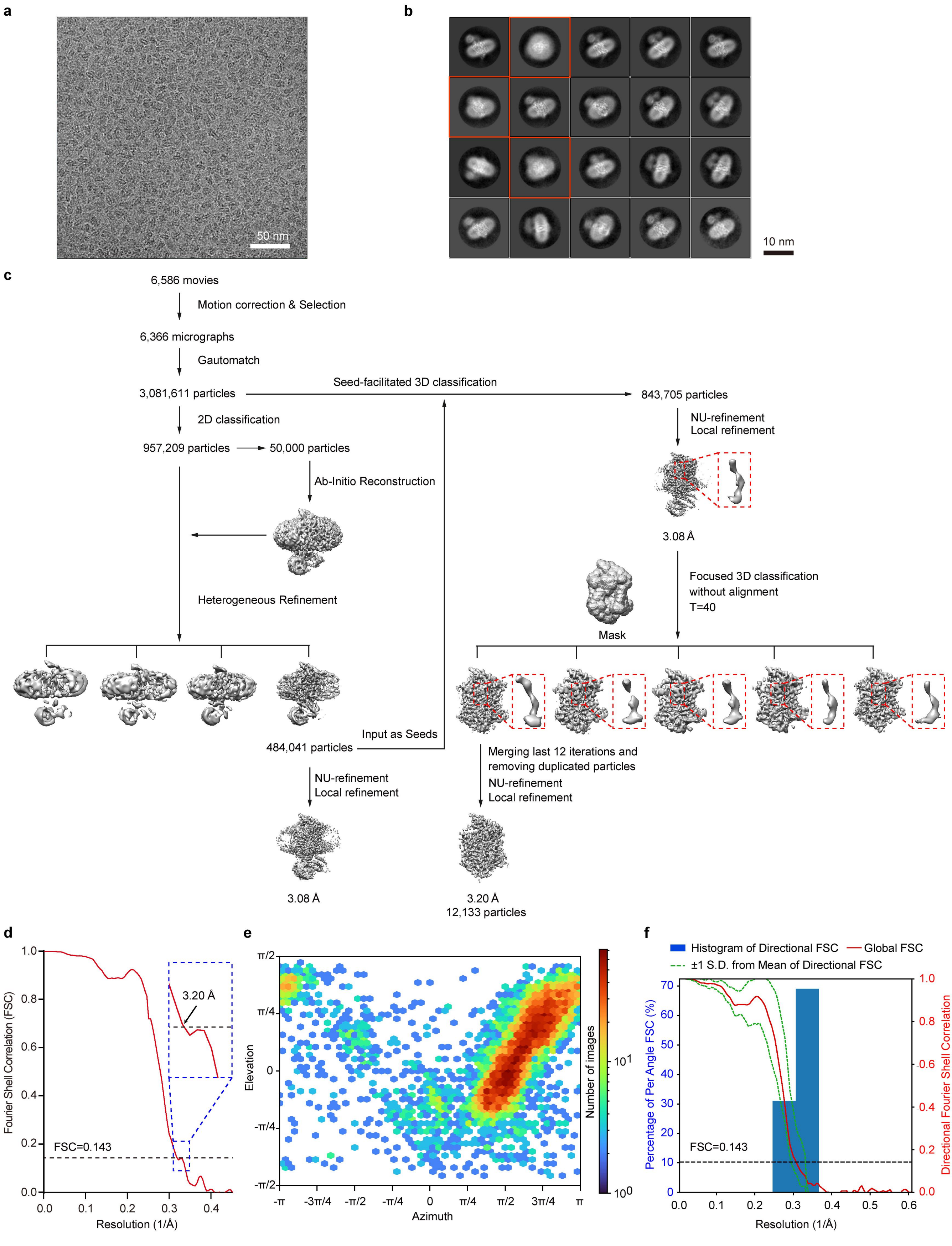

Supplementary Fig. 2 | Cryo-EM data processing of hSGLT1. a, Cryo-EM micrograph of hSGLT1. Experiment was repeated independently more than three times with similar results. **b,** No-alignment 2D class averages of the particles used in the final reconstruction. The 2D class averages were sorted by particle number from more to less. The top views of hSGLT1 were highlighted in red box. **c,** Flowchart of image processing. **d,** The gold-standard FSC curve for the cryo-EM map. **e,** Euler angle distribution of all particle images that contributed to the final 3D map. **f,** Histogram of directional FSC curves. Individual 1D FSC curves are compiled into the 3D FSC and represented within a histogram. The spread of the directional resolutions defined by plus and minus one standard deviation from the mean of the directional resolutions. Source data are provided as a Source Data file.

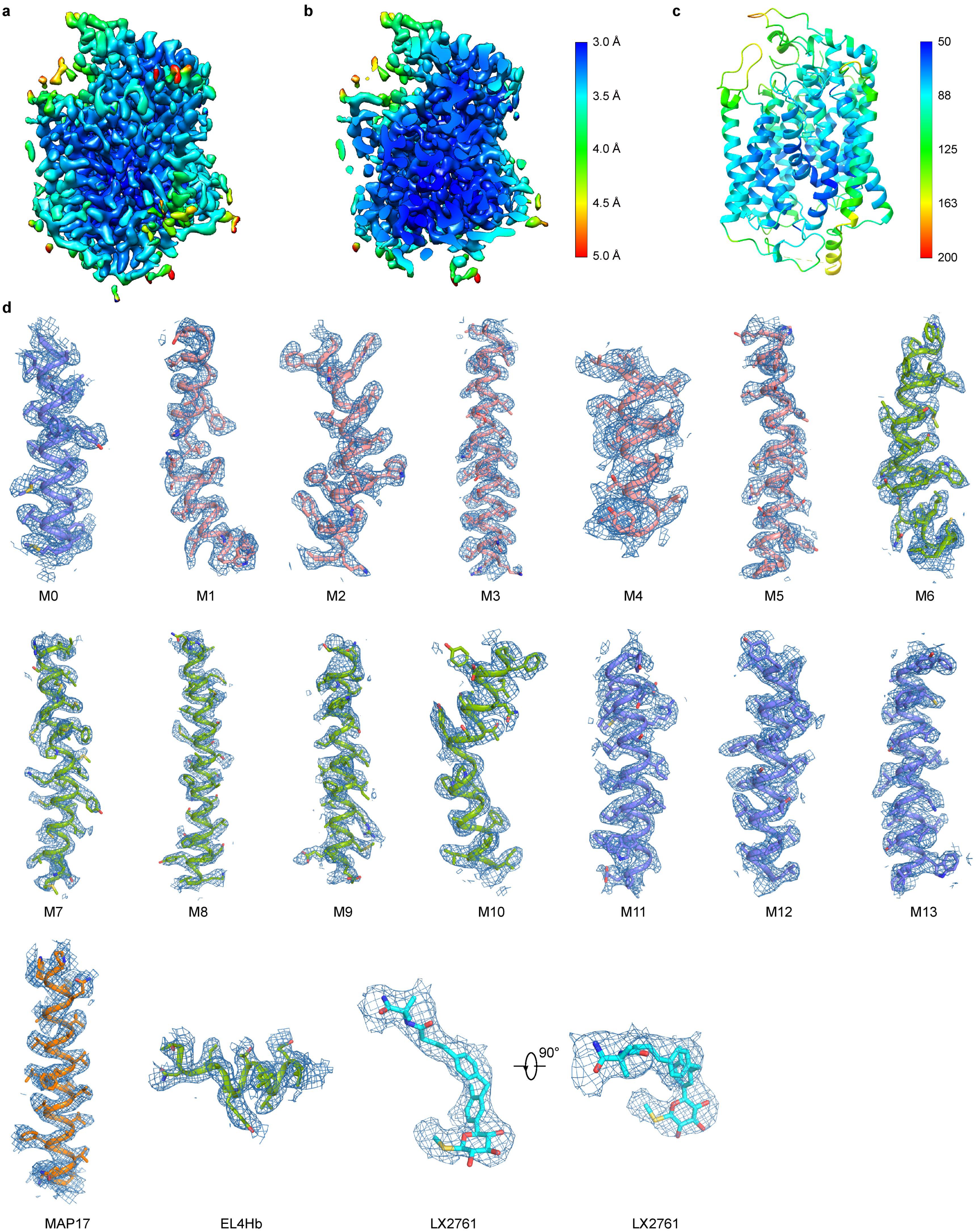

Supplementary Fig. 3 | Cryo-EM densities and structural models of hSGLT1. a, Local resolution of hSGLT1 estimated with cryoSPARC. **b,** The cut-open view of local resolution distribution of hSGLT1. **c,** Thermal parameter (B-factor) distribution in hSGLT1. **d,** The PDB models of SGLT1 are overlaid with the electron density shown in blue meshes.

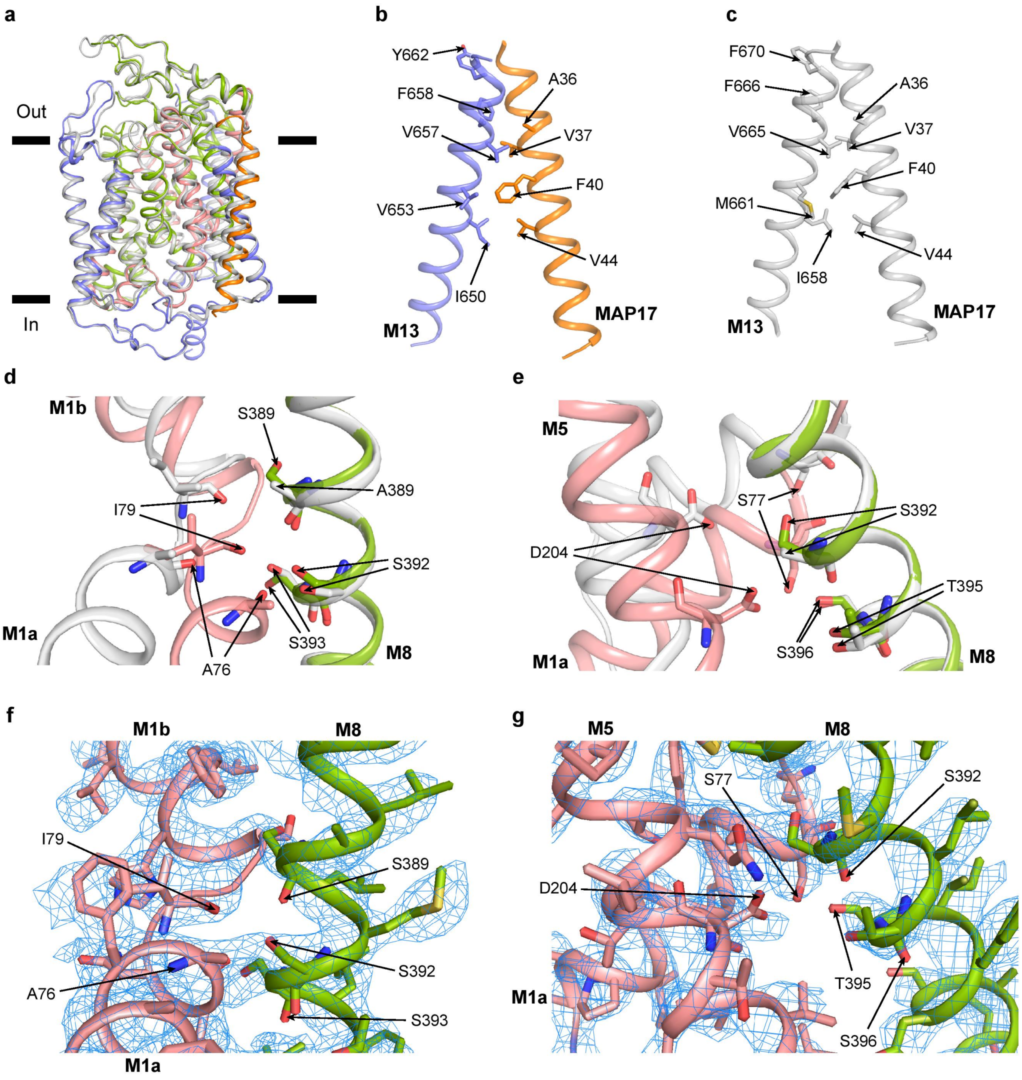

Supplementary Fig. 4 | Structural comparison of hSGLT1 and hSGLT2. a, Superposition of hSGLT1$_{\text{outward-open}}$ (colored) and hSGLT2$_{\text{outward-open}}$ (grey, PDB ID: 7VSI). **b,** The interactions between MAP17 and M13 of hSGLT1. **c,** The interactions between MAP17 and M13 of hSGLT2. **d,** Structural alignment of sodium-binding site (Na2 site) of hSGLT1$_{\text{outward-open}}$ (colored in pink and green) and hSGLT1$_{\text{inward-open}}$ (colored in grey). **e,** Structural alignment of sodium-binding site (Na3 site) of hSGLT1$_{\text{outward-open}}$ (colored in pink and green) and hSGLT1$_{\text{inward-open}}$ (colored in grey). **f,** Electron density of sodium-binding site (Na2 site) of hSGLT1. **g,** Electron density of sodium-binding site (Na3 site) of hSGLT1.

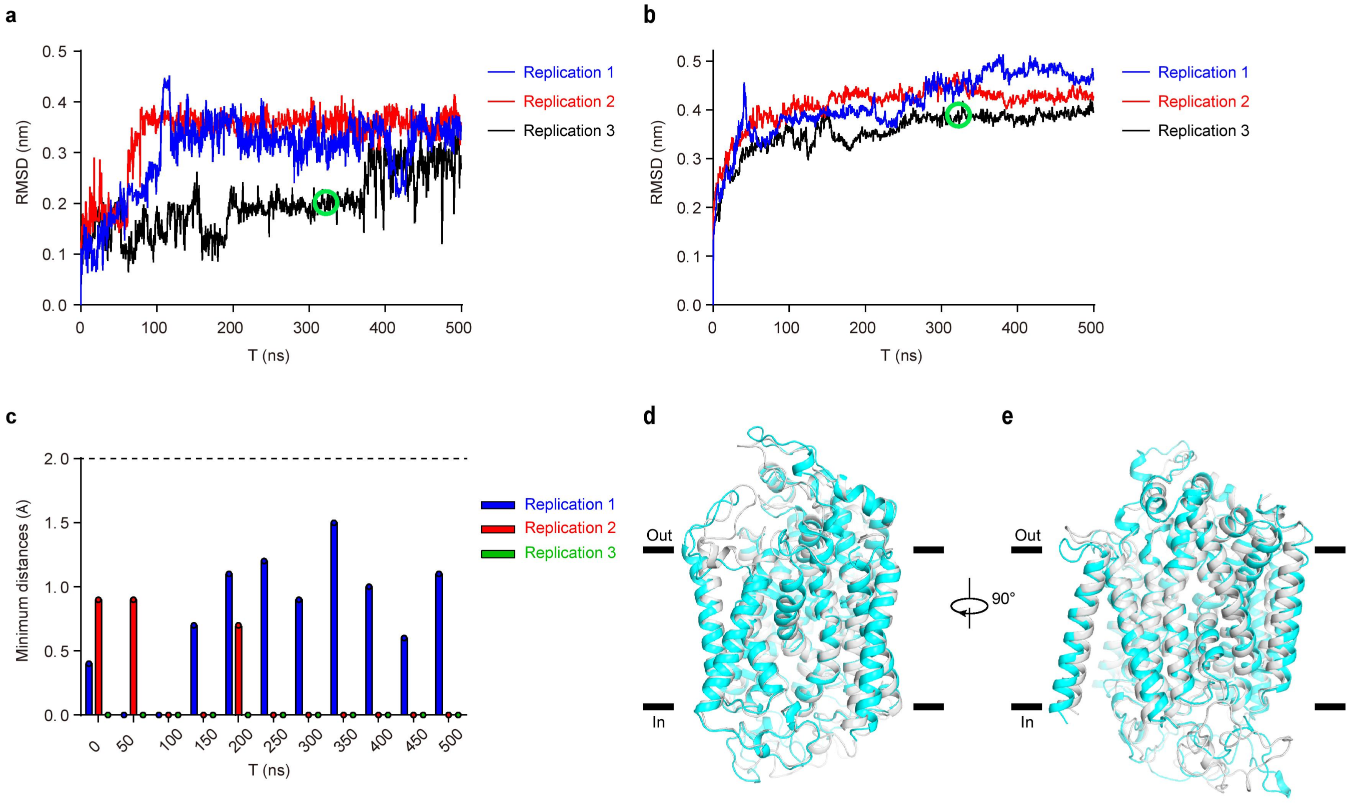

Supplementary Fig. 5 | All-atom molecular dynamics simulations of hSGLT1 with Mizagliflozin. a, RMSD (Root Mean Square Deviation) of the Mizagliflozin during the 500 ns simulation. The RMSD values are calculated with the protein in trajectories superimposed on the protein in the first frame. The representative simulation frame used for structural alignment in Fig. 3a-b is indicated in green circle. **b,** RMSD of the hSGLT1-MAP17 during the 500 ns simulation. The representative simulation frame used for structural alignment in Fig. 3a-b is indicated in green circle. **c,** Minimum distances between V157 of SGLT2 and mizagliflozin during the MD simulation. SGLT2 was aligned to the SGLT1-mizagliflozin complex sampled every 50 ns in trajectory during simulation. Hydrogen atoms were included in the distance measurements. Distances that are no more than zero were shown as zero. The horizontal dash line represent the twice of the radius of hydrogen (2Å). **d,** Superimposition of the starting simulation frame and the representative simulation frame. The representative simulation frame is shown in cyan. The starting simulation frame is shown in grey. **e,** A 90° rotated view of **d**. Source data are provided as a Source Data file.

Supplementary Table 1
Chemical structures and inhibition activities of various hSGLT inhibitors

Inhibitor	Chemical structure	IC_{50} (nM)[a]		hSGLT2 selectivity fold (vs hSGLT1)
		hSGLT1	hSGLT2	
Empagliflozin	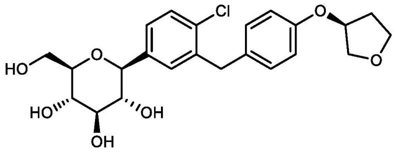	8,300	3.1	~2,700
Dapagliflozin	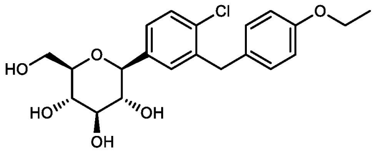	1,400	1.2	~1,200
Sotagliflozin	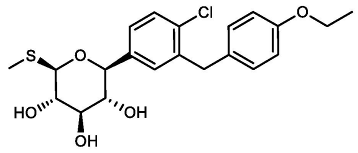	36	1.8	~20
LX2761	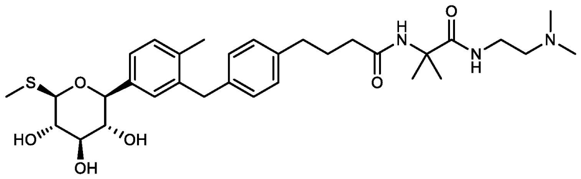	2.2	2.7	~0.8
Mizagliflozin	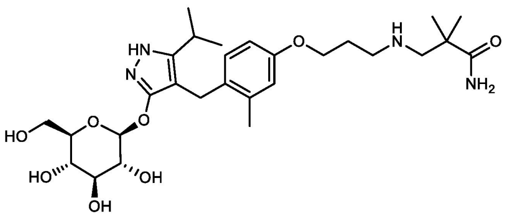	27	8,170	~0.003

[a] Numbers are from Ref.10.

Supplementary Table 2
Cryo-EM data collection, refinement and validation statistics

	hSGLT1-MAP17 complex with LX2761 bound (EMD-32617) (PDB 7WMV)
**Data collection and processing**	
Magnification	165,000×
Voltage (kV)	300
Electron exposure (e–/Å^2)	37.6
Defocus range (μm)	-1.5 to -1.8
Pixel size (Å)	0.821
Symmetry imposed	*C1*
Initial particle images (no.)	3,081,611
Final particle images (no.)	12,133
Map resolution (Å)	3.20
FSC threshold	0.143
Map resolution range (Å)	200-3.20
**Refinement**	
Initial model used (PDB code)	7VSI
Model resolution (Å)	3.20
FSC threshold	0.143
Model resolution range (Å)	200-3.20
Map sharpening *B* factor (Å^2)	-107.3
Model composition	
Non-hydrogen atoms	4,899
Protein residues	636
Ligands	1
B factors (Å^2)	
Protein	95.25
Ligand	89.50
R.m.s. deviations	
Bond lengths (Å)	0.007
Bond angles (°)	1.242
Validation	
MolProbity score	2.11
Clashscore	9.72
Poor rotamers (%)	2.14
Ramachandran plot	
Favored (%)	94.92
Allowed (%)	4.92
Disallowed (%)	0.16

Supplementary Table 3

The potencies of SGLT inhibitors on various hSGLT1 and hSGLT2 constructs

hSGLT1/hSGLT2 constructs	LX2761		Mizagliflozin	
	$LogIC_{50}$[a]	IC_{50} (nM)	$LogIC_{50}$[a]	IC_{50} (nM)
hSGLT1 constructs				
WT	0.209 ±0.296	1.62	1.646 ± 0.031	44.26
L274A	0.642 ± 0.145	4.38		
D454A	1.165 ± 0.081	14.62		
A160V			2.404 ± 0.141	253.99
hSGLT2 constructs				
WT			3.949 ± 0.035	8891.42
V157A			2.945 ± 0.138	881.72

[a] Data are expressed as $logIC_{50}$ ± standard deviations; n = 3 biologically independent experiments.

Supplementary Table 4
Primers and protein sequences used in this study

Primer Name	Sequence
hSGLT1-A160V-F	agatctcggTagacatcttctc
hSGLT1-A160V-R	aagatgtctAccgagatcttgg
hSGLT1-L274A-F	cgggagacGCcccatggcctgg
hSGLT1-L274A-R	ggccatgggGCgtctcccgtga
hSGLT1-D454A-F	aactcttcgCttacatccagtc
hSGLT1-D454A-R	tggatgtaaGcgaagagttgcc
hSGLT1-H525A-F	gtggggtgGCctacttgtactt
hSGLT1-H525A-R	tacaagtagGCcaccccacaga
hSGLT1-H525F-F	gtggggtgTTctacttgtactt
hSGLT1-H525F-R	tacaagtagAAcaccccacaga
hSGLT2-V157A-F	accaagatctcaGCggacatgttctccgga
hSGLT2-V157A-R	gagaacatgtccGCtgagatcttggtgaa

hSGLT1$_{GFP}$ Sequence:

MDSSTWSPKTTAVTRPVETHELIRNAADISIIVIYFVVVMAVGLWAMFSTNRGTVGGFFLAGRSMVWWPIGASLFASNIGSGHFVGLAGTG
AASGIAIGGFEWNALVLVVVLGWLFVPIYIKAGVVTMPEYLRKRFGGQRIQVYLSLLSLLLYIFTKISADIFSGAIFINLALGLNLYLAIFLLL
AITALYTITGGLAAVIYTDTLQTVIMLVGSLILTGFAFHEVGGYDAFMEKYMKAIPTIVSDGNTTFQEKCYTPRADSFHIFRDPLTGDLPWPG
FIFGMSILTLWYWCTDQVIVQRCLSAKNMSHVKGGCILCGYLKLMPMFIMVMPGMISRILYTEKIACVVPSECEKYCGTKVGCTNIAYPTL
VVELMPNGLRGLMLSVMLASLMSSLTSIFNSASTLFTMDIYAKVRKRASEKELMIAGRLFILVLIGISIAWVPIVQSAQSGQLFDYIQSITSYL
GPPIAAVFLLAIFWKRVNEPGAFWGLILGLLIGISRMITEFAYGTGSCMEPSNCPTIICGVHYLYFAIILFAISFITIVVISLLTKPIPDVHLYRLC
WSLRNSKEERIDLDAEEENIQKMSKGEELFTGVVPILVELDGDVNGHKFSVRGEGEGDATIGKLTLKFICTTGKLPVPWPTLVTTLTYGVQC
FSRYPDHMKRHDFFKSAMPEGYVQERTISFKDDGKYKTRAVVKFEGDTLVNRIELKGTDFKEDGNILGHKLEYNFNSHNVYITADKQKNG
IKANFTVRHNVEGSGHHHHHHHHADRKAAVSHWQQSSGLVPRGSGWSHPQFEKGSGDYKDDDDKGSGWSHPQFEKGSGDGSVQLADH
YQQNTPIGDGPVLLPDNHYLSTQTKLSKDPNEKRDHMVLHEYVNAAGITEGPKETIEIETQVPEKKKGIFRRAYDLFCGLEQHGAPKMTEE
EEKAMKMKMTDTSEKPLWRTVLNVNGIILVTVAVFCHAYFA*

MAP17$_{nb}$ Sequence:

MSALSLLILGLLMAVPPASCQQGLGNLQPWMQGLIAVAVFLVLVAIAFAVNHFWCQEVALVESGGALVQPGGSLRLSCAASGFPVNRYSMR
WYRQAPGKEREWVAGMSSAGDRSSYEDSVKGRFTISRDDARNTVYLQMNSLKPEDTAVYYCNVNVGFEYWGQGTQVTVS*
